# Editorial Notes

**Published:** 1938

**Authors:** 


### Editorial Notes

**The Standard of Living in Bristol.**

THE Report of the Colston Research Society for 1935–36 announced that a Fund had been established for making a Social Survey of Bristol, and that Mr. Herbert Tout, M.A., had been appointed to a Colston Research Fellowship for the purpose of directing the Survey. A preliminary report has just been published under the title *The Standard of Living in Bristol*, which gives a brief outline of what the full report, now nearing completion, will describe in much greater detail. This pamphlet confines itself to findings on the standard of living, the aspect of the Survey which is probably of most general interest. The Survey is concerned only with that section of the people with incomes below £250 a year, which is estimated to comprise nine-tenths of the population. The method is to adopt a minimum standard of needs for the unit of the family, and by comparing income to this standard to express in terms of percentage the standard of living of the population.

To quote figures from the pamphlet in any abridged form is misleading. It is sufficient here to say that on the nutrition side the standard of needs assumes the minimum dietary recommended by the British Medical Association report of 1933, and that no one is likely to dispute the conclusion that the standard adopted is a very reasonable interpretation of the poverty line. The medical profession are probably more aware of the extent of poverty than most classes of the community, but it must surely come as a shock to the citizens of Bristol to learn that in the Survey area about 11,000 families are living below this poverty line; that this number includes one in five of all children under 14; and that of those above the line some 21,000 families are separated from it by so small a margin that expenditure on recreation, tobacco or drink is liable to bring them below.

It is interesting to note how small was the opposition to what must have been a somewhat delicate investigation. About 93 per cent. of the families approached gave the desired information, a tribute to the tact with which the inquiry was conducted.

Mr. Tout has been notably successful in his task of compressing the data into sixty-four pages, and at the same time making such a clear and intelligible summary of the findings. The scientific approach to social problems is still in its infancy. This cold and dispassionate presentation of scientifically established facts is more painfully convincing than any appeal of charity, and should do far more to bring home to public attention the urgency and the immensity of the problem.

**Benger's Food, New Factory.**

IN the heart of Cheshire, amid picturesque country surroundings, stands the new home of Benger's Food Ltd. It is at Holmes Chapel—some twenty-five miles south of Manchester. This new factory has been built to provide accommodation for the manufacture of new pharmaceutical and medical products. It stands in what will shortly be spacious gardens at the front and at one side, and sports ground on the other side, and has been built as a square bounding a quadrangle which will be made into a lawn and rose garden. The main frontal portion of the building contains on the ground floor: stock room, despatch and printing departments, and on the upper floor: executive and general offices, laboratory and boardroom. The manufacturing processes and packing of Benger's Food and other specialities are carried out in the rear portion of the factory. Striking features of the manufacturing section are the glazed white tiles, which surface the walls from ground to ceiling—an introduction which makes for brightness and cleanliness in any building—the loftiness and spaciousness of all departments, the modern decorations and lighting, and the red tiled floor which is laid throughout.

Messrs. Benger's Food Ltd. will welcome the visit of members of the medical profession who may be passing, at any time between the hours of ten and twelve, and two and four o'clock. Holmes Chapel is on the main road from the Potteries to Manchester.

